# Efficient Sensitivity Based Reconstruction Technique to Accomplish Breast Hyperelastic Elastography

**DOI:** 10.1155/2018/3438470

**Published:** 2018-11-25

**Authors:** Maryam Mehdizadeh Dastjerdi, Ali Fallah, Saeid Rashidi

**Affiliations:** ^1^Department of Biomedical Engineering, Amirkabir University of Technology (AUT), Tehran 15875-4413, Iran; ^2^Faculty of Medical Sciences and Technologies, Science and Research Branch, Islamic Azad University, Tehran 1477893855, Iran

## Abstract

Hyperelastic models have been acknowledged as constitutive equations which reliably model the nonlinear behaviors observed from soft tissues under various loading conditions. Among them, the Mooney-Rivlin, Yeoh, and polynomial models have been proved capable of accurately modeling responses of breast tissues to applied compressions. Hyperelastic elastography technique takes advantage of the disparities between hyperelastic parameters of varied tissues and the change in hyperelastic parameters in pathological processes. The precise reconstruction of hyperelastic parameters of a completely unknown pathology in the breast in a noninvasive and nondestructive way using the ultrasound elastography has been scrutinized in this paper. In the ultrasound elastography, tissue displacement field is extracted from radio frequency signals or images recorded using the ultrasound medical imaging system; hence the exact displacement field might not be obtained. Our results indicate that the parameters estimated by manipulating the iterative sensitivity-matrix based method converge to tissue's real hyperelastic parameters providing appropriate parameters are assigned to the hypothetical hyperelastic and regularization parameters. Iterative methods have therefore been proposed to compute proper hypothetical hyperelastic and regularization parameters. Accurate estimates of hyperelastic parameters of obscure breast pathology have been achieved even from imprecise measurements of displacements induced in the tissue by the ramp excitation.

## 1. Introduction

In the past two decades, substantial efforts have been made to bring the novel promising imaging approach “elastography” proposed by Ophir et al. [1] into clinical use. In contrast to the common medical imaging methods, elastography techniques could noninvasively provide qualitative or quantitative information on mechanical characteristics of biological tissues [2, 3]. Distinct perceptible changes in the mechanical attributes of a biological soft tissue in varying pathologies yield the noninvasive detection and classification of its lesions using elastography techniques. Among them, ultrasound elastography approaches, which benefit from the superiority of conventional ultrasound (US) as being a safe, easy-to-use, inexpensive, nondestructive, noninvasive, widely available, and versatile medical imaging system, have received considerable attention.

In general, the practicable US elastography methods are classified on the basis of measured tissue-correlated physical quantities into [4]:

1. Strain Imaging: which is capable of qualitatively imaging Young's modulus,* E*, of the tissue by taking the response of tissue to a quasistatic load into consideration.
2. Shear Wave Imaging (SWI): which is capable of measuring shear wave speed,* c*_s_, in the tissue (or its Young's modulus,* E*,) by taking the dynamic response of tissue to a mechanical vibration or acoustic radiation force into account.

Various praxes, particularly biomechanical characterization and imaging, have attributed the significant rise in the proposal of techniques for estimating linear and nonlinear elastic parameters of materials, i.e., for solving inverse problems in elasticity. Invaluable surveys of the relevant techniques have been provided by

- Bonnet and Constantinescu [5],
- Hajhashemkhani et al. [6],
- Guchhait and Banerjee [7].

The aforementioned reviews have respectively discussed:

- The formulations and solution methods, specifically the finite element and boundary element based methods, which are pertinent to general numerical methods for solving unspecialized elasticity problems on complex configurations, namely, least-squares functionals, adjoint solutions, and error in constitutive equation (ECE) based cost functions.
- Momentous approaches for characterizing mechanical properties, i.e., hyperelastic parameters, of biological soft tissues and rubber-like materials with regard to the testing methods utilized, e.g., indentation tests, pipette aspiration experiments, propagation of elastic waves in the medium, inflation experiments, equibiaxial tensions, and in vivo experiments.
- Prime elastic parameter estimation techniques involving linear elastic regime and geometrically or materially nonlinear forward model, for example, least-square based cost functionals, sensitivity based tactics, and integral approaches.

Besides the clinical applications, the introduction of numerous innovative strategies to estimate the nonlinear elastic parameters of materials or to reduce their computational costs, for instance,

- virtual fields method (VFM) [8, 9],
- subdomain inverse finite element technique [10],
- pointwise identification approach [11],
- gradient-based quasi-Newton minimization, adjoint, and continuation strategies [12],
- the minimization-based reconstruction technique enhanced through material parameter grouping and user-supplied gradients of the objective function together with a nonlinear adjoint method [13],

to name but a few, infers the importance of the subject of study.

As regards our research focus, in brief, a least-square based cost functional was primarily applied by Iding et al. in 1974 [14] to estimate hyperelastic parameters of homogeneous materials. With respect to the geometrically nonlinear response of soft tissues at large deformations, i.e., the nonlinearity of the strain-displacement relation, an integral approach has been proposed by Skovoroda et al. [15] to reconstruct the elasticity distribution of soft tissue. Sensitivity based approaches have been applied formerly by Gendy and Saleeb [16] and recently by Hajhashemkhani and Hematiyan [6, 17, 18] to estimate hyperelastic parameters of rubber-like materials and soft tissues. The slope-variation method and Nelder-Mead algorithm have been applied by O'Hagan and Samani [19, 20] and Naini et al. [21] to evaluate hyperelastic parameters of abnormal breast and deflated lung tissues.

With the aim of diagnosing breast cancers through reconstructing the spatial distributions of linear and nonlinear elastic parameters in patients with benign and malignant tumors [22, 23], the inverse nonlinear elasticity problem has been altered into a minimization problem by Gokhale et al. [24]. The gradient-based quasi-Newton optimization method has been applied to minimize the cost function in consideration of the spatial distribution of material properties. The adjoint elasticity equations and continuation (in the material properties) scheme have been employed to calculate the gradient in reasonable time.

The application of US elastography technique to reconstruct nonlinear elastic constants of normal and abnormal breast tissues has been intended in this paper. The response of breast tissue, with and without the lesion, to a ramp stimulus (with low rate of increase in the applied load to ignore the inertia effect) has been simulated with the help of the finite element (FEM) software, Abaqus FEA. Two iterative methods founded on the stress-strain relation and sensitivity matrix have been, respectively, applied to estimate proper hypothetical and precise real hyperelastic parameters for the unknown tissues from limited displacement quantities in the tissues.

The approximate estimation of tissue displacement fields from the simulated US radio frequency (RF) signals, using the cross-correlation algorithm, impelled us to determine proper regularization parameter to converge to the real hyperelastic parameters of the tissue. An analogous iterative tactic has therefore been employed to compute proper regularization parameters. The decrease in the errors of elastic parameters estimated for the tumor, by comparing with the real elastic parameter estimated for the tumor, is the essence of the proposed iterative methods which lead to appropriate hypothetical and accurate real hyperelastic constants and suitable regularization parameter.

## 2. Materials and Methods

### 2.1. Hyperelastic Constitutive Models

The results of multitude experimental scrutinizations of the behaviors of biological soft tissues, such as the breast and its lesions, have confirmed their nonlinear responses to applied stresses [25–27]. The researchers in the continuum mechanics work towards constructing mathematical models (mathematical equations) which could [28–30]

- realistically represent the behavior of the understudy material;
- assess the material's response to the applied load;
- differentiate materials.

Hyperelastic constitutive models have made us competent at representing the nonlinear elastic responses of soft tissues to (large) strains. The constitutive theory of hyperelastic materials regards both the nonlinearity in the material behavior and considerable changes in the shape of material [31–33].

The strain energy density function, aka stored energy function, characterizes the energy absorbed by the homogeneous material in consequence of its deformation. Certain strain energy density functions are utilized to describe hyperelastic materials. As regards the deformation gradient tensor,** F**, (1) defines the strain energy function, *W*, as a function of** F**,

(1) $$\begin{array}{c} W=W\left(\;F\;\right). \end{array}$$

The invariants of deformation,** F**, aka strain invariants of deformation, make mapping the area and volume between the deformed and reference configurations possible. The first, second, and third invariants of deformation, *I*_1_, *I*_2_, and *I*_3_, are computed by the use of (2) for unconstrained isotropic elastic materials,

(2) I1=trF=F11+F22+F33,I2=12FijFji−FiiFjj,I3=detF=J.

The left and right Cauchy-Green deformation tensors,** B** and** C**, are computed using

(3) B=FFT,

(4) C=FTF.

The principal invariants of** B** and** C** are calculated as follows:

(5) I1B=trB,I2B=12I1B2−trB2,I3B=det⁡B≡det⁡F2,

(6) I1C=trC,I2C=12I1C2−trC2,I3C=detC≡det⁡F2.

As represented for** B** in (7), for incompressible materials, different sets of principal invariants of** B **and** C** are applied,

(7) I−1B=I1BJ2/3,I−2B=I2BJ4/3,Jel=detB.

Equation (8) specifies the Cauchy stress tensor of an unconstrained isotropic elastic material in terms of strain invariants, *I*_1_, *I*_2_, and *I*_3_,

(8) σ=α0I+α1B+α2B2,α0=2I31/2∂W∂I3,α1=2I3−1/2∂W∂I1+I1∂W∂I2,α2=−2I3−1/2∂W∂I2.

The comprehensive compilations of the relations associated with the hyperelasticity theory have been provided by Bower [34], Felippa [35], and Holzapfel and Ogden [36]. On account of the unity of *I*_3_ (and therefore *α*_0_=0), the Cauchy stress tensor relationship for incompressible materials simplifies to

(9) $$\begin{array}{c} \sigma =-pI+2\frac{\partial W}{\partial I_{1}}B+2\frac{\partial W}{\partial I_{2}}\left(\;I_{1}B-B^{2}\;\right), \end{array}$$

where *p* refers to the arbitrary hydrostatic pressure, i.e., the Lagrange multiplier which compels the incompressibility constraint.

With the aim of accurately modeling the nonlinear elastic behaviors observed from soft tissues, a multitude of strain energy functions have been introduced in the literature. The functions have been defined in terms of

- strain invariants, for instance, *I*_1_, *I*_2_, and *I*_3_ or ${\bar{I}}_{1}$, ${\bar{I}}_{2}$, and* J,*
- hyperelastic parameters, *C*_*ij*_.

The functions extend from the well-known long-established Neo-Hookean and Mooney-Rivlin models (originated respectively by Treloar [37] in 1943, and Rivlin and Saunders [38] in 1951) to the recently inaugurated models, as the ones proposed by Nolan et al. [39] in 2014, Chen et al. [40] in 2015, and Carniel and Fancello [41] in 2017. On the basis of the outcomes of several investigations, which have been referred in Section 3.1, three strain energy functions,

- Mooney-Rivlin model (special first-order form of generalized Rivlin model),
- Yeoh model,
- Second-order generalized Rivlin model, aka (second-order) polynomial model,

could precisely model the behavior of breast tissues. The mentioned models are, respectively, formulated (in simplified forms since* J*=1 for incompressible tissues) by

(10) W=C10I1−3+C01I2−3,

(11) W=C10I1−3+C20I1−32+C30I1−33,

(12) W=C10I1−3+C01I2−3+C20I1−32+C11I1−3I2−3+C02I2−32.

While a uniaxial stress, **σ**, is applied to the medium, the deformation gradient tensor,** F**, is computed using (13), where every *λ*_*i*_ (*i*=1,2,3) refers to the principal stretch parallel to one of the coordinate axes,

(13) $$\begin{array}{c} F=\left[\begin{array}{ccc} \lambda_{1} & 0 & 0\\ 0 & \lambda_{2} & 0\\ 0 & 0 & \lambda_{3} \end{array}\right]. \end{array}$$

The principal stretches determine the principal invariants, *I*_1_, *I*_2_, and *I*_3_, as represented,

(14) I1=λ12+λ22+λ32,I2=λ12·λ22+λ22·λ32+λ32·λ12,I3=λ12·λ22·λ32.

If the uniaxial stress, **σ**, applied to the medium is considered in line with the first coordinate axis, (15) defines the uniaxial strain, **ε**, produced in the medium due to the applied stress,

(15) $$\begin{array}{c} \varepsilon =\lambda -1, \end{array}$$

where ***λ*** represents the parallel stretch. Through assuming (1) the incompressibility of the medium (i.e., *I*_3_=1) and (2) the equivalence of deformations in the two other coordinate axes, the Cauchy stress tensor equation for an incompressible medium (9) is simplified to [42, 43]

(16) $$\begin{array}{c} \sigma =2\left(\;\lambda^{2}-\lambda^{-1}\;\right)\left(\;\frac{\partial W}{\partial I_{1}}+\frac{1}{\lambda }\frac{\partial W}{\partial I_{2}}\;\right). \end{array}$$

Regarding the above explanations, (17), (18), and (19) represent the Cauchy stress-stretch relations of the Mooney-Rivlin, Yeoh, and second-order polynomial models, respectively,

(17) σ=2λ2−λ−1C10+λ−1C01,

(18) σ=2λ2−λ−1C10+2C20λ2+2λ−1−3+3C30λ2+2λ−1−32,

(19) σ=2λ2−λ−1C10+C01λ−1+2C20λ2+2λ−1−3+2C022λ+λ−2−3+3C11λ−1−λ−1+λ−2.

In consideration of (15), the following stress-strain relationships have been, respectively, achieved for the Mooney-Rivlin, Yeoh, and polynomial models,

(20) σ=21+ε2−1+ε−1C10+1+ε−1C01,

(21) σ=21+ε2−1+ε−1C10+2C201+ε2+21+ε−1−3+3C301+ε2+21+ε−1−32,

(22) σ=21+ε2−1+ε−1C10+C011+ε−1+2C201+ε2+21+ε−1−3+2C0221+ε+1+ε−2−3+3C111+ε−1−1+ε−1+1+ε−2.

### 2.2. Estimation of Hyperelastic Parameters

The differences between the hyperelastic constants of normal and abnormal breast tissues, as thoroughly discussed in [19, 20, 44] for numerous ex vivo breast tissue samples, make the detection and identification of breast tumors through their hyperelastic parameters feasible. With the purpose of precisely estimating the parameters of the selected hyperelastic models, namely,

- the Mooney-Rivlin parameters, i.e., *C*_10_ and *C*_01_ of (10),
- the Yeoh parameters, i.e., *C*_10_, *C*_20_, and *C*_30_ of (11),
- the polynomial parameters, i.e., *C*_10_, *C*_20_, *C*_11_, *C*_02_, and *C*_01_ of (12),

for normal and pathological breast tissues, two iterative algorithms have been appraised. The algorithms are founded on

- the stress-strain relationship and sensitivity matrix, which have been formed on the basis of the relation of the selected hyperelastic model, as explained in Sections 2.1 and 2.2,
- the noninvasive measurement of displacement and strain fields in the understudy tissue from the recorded US RF signals and images.

Two types of software, MATLAB® (The MathWorks, Inc., Natick, Massachusetts, USA) and a FEM software, for instance, Abaqus FEA (Dassault Systèmes Simulia Corp., Johnston, RI, USA), should be bilaterally connected to make the automatic iterations of the propounded algorithms possible.

At first, the recommended iterative algorithm which progressively provides approximate estimates of hyperelastic parameters of an undiagnosed tissue is described in this section with regard to

1. the explanations and equations mentioned in Section 2.1, particularly the ones associated with the relations between stress sets, strain sets, and the parameters of the Mooney-Rivlin, Yeoh, and polynomial hyperelastic models;
2. the availability of precise information on mechanical characteristics, i.e., linear or nonlinear elastic parameters, of normal mediums that surround the tissue. To the best of our knowledge, at least, the elastic parameters of almost all healthy soft tissues, mainly measured through minute ex vivo experiments, have been reported in the literature. For several soft tissues, in particular the breast, as mentioned previously, we could benefit from the accessibility to precise information on their nonlinear mechanical constants.

The above-mentioned algorithm could be described in the following steps:

(a) The unknown tissue, i.e., tumor, and its surrounding mediums are imaged before/while responding to a ramp excitation (with low rate of increase in the applied load to negate the inertia effect) for a period of time by the employment of a clinical US imaging system.
(b) The registered precompression US images, loading specifications, and boundary conditions are delicately regarded to accurately simulate the tumor and its adjacent mediums with the help of the FEM software, Abaqus FEA. Further explanation of the simulation strategies has been provided at the end of the section.
(c) In the simulated specimen, the values of 1 Pa and 0.5 are, respectively, assigned to the elastic modulus and Poisson's ratio of the tumor to simulate an equivalent elastic tumor.
(d) The displacement fields at some consecutive step times are extracted
  (i) for the tumor, from the recorded US RF signals or images, for instance, by the use of the cross-correlation algorithm; i.e., the exact displacement fields in the tumor at some instants are computed;
  (ii) for the simulated elastic tumor, employing the FEM software, Abaqus FEA.
(e) The real elastic modulus of the tumor, *E*_real_, is computed using ([17, 18, 45])
  (23) $$\begin{array}{c} E_{real}=\frac{D^{T}D}{D^{T}Y_{real}}, \end{array}$$
where **Y**_real_ and** D**, respectively, represent the axial displacement values of some points of the tumor and elastic tumor at the specified moments.
(f) The tumor strain field could be roughly approximated from the displacement measurements. The estimated elastic modulus for the tumor, *E*_real_, is used to calculate a set of stress values, **σ**, from a set of strain values, **ε** (which is formed with regard to the strain field in the tissue), through the linear elasticity relation, (24), known as Hook's law,
  (24) $$\begin{array}{c} \sigma =E_{real}\varepsilon . \end{array}$$
In view of the obtained results, a set of arbitrary strain values could also be considered, although the selection of strain set based on the available information leads to the significant decrease in the number of iterations.
(g) The parameters of the elected hyperelastic models, namely, the Mooney-Rivlin, Yeoh, and polynomial models, are computed using the formed stress and strain sets and the relation between stress, strain, and the parameters of a hyperelastic model, as represented in Section 2.1, i.e., (20), (21), and (22), for instance, with the help of regression algorithms of the MATLAB software.
(h) The elastic parameter for the simulated tumor, *E*_sim_, is calculated after assigning the estimated hyperelastic parameters to the tumor and measuring the axial displacement values of the appointed points at the chosen step times, **Y**_sim_, by substituting **Y**_sim_ for **Y**_real_ in (23). The error of the computed elastic parameter, *E*_sim_, while it is compared with *E*_real_, i.e., the real elastic modulus calculated for the tumor, is used to evaluate the estimated hyperelastic parameters for the tumor. The assumption of the unavailability of initial information about the tumor and its mechanical characteristics has compelled us to consider the minimum of the errors of the estimates of tumor elastic modulus the criterion, as represented,
  (25) Esimk−Ereal<Esimi−Ereal,i=1,2,…,k−1,k+1,…,M
where *M* is the number of iterations of the algorithm.
(i) Through correctly modifying the strain set specified in step (f) and iterating the algorithm from this step, the estimated hyperelastic parameters could even converge to the real hyperelastic parameters of the tumor. A strain set that is slightly greater or less than the strain values, which are roughly computed from the tissue displacement measurements, would be the most appropriate choice; therefore, in this step, the strain quantities should be, respectively, decreased or increased.

The errors of the elastic parameters estimated for the tumor might not decrease below the defined tolerance value, specifically while the strain set is elected arbitrary. The iterative algorithm based on the sensitivity matrix, as defined by Hajhashemkhani and Hematiyan [17, 18], would be the right choice to converge to accurate estimates of tumor hyperelastic parameters. The attained results indicate that the selection of suitable initial hyperelastic parameters, which could be obtained by the use of the proposed iterative method, is imperative to converge to precise estimates of tumor hyperelastic parameters through the sensitivity-matrix based algorithm; otherwise, the algorithm might approach the local minima.

The following steps specify the sensitivity-matrix based algorithm which has been applied to estimate the parameters of the Mooney-Rivlin, Yeoh, and polynomial hyperelastic models:

(j) Similar to the previous algorithm, the US images and RF signals recorded from the mediums which have surrounded the obscure tissue are considered. The data are being registered for a time period before/while the mediums are responding to a ramp excitation (with low incremental rate to annul the inertia) by the use of the clinical US imaging system.
(k) By dint of the FEM software, the tumor and its adjacent mediums are meticulously simulated considering the saved precompression US images, loading specifications, and boundary conditions. The simulation tactics have further been explained at the end of the section.
(l) The axial displacement values of the specified points of the tumor at the determinate step times, **Y**_real_, are extracted from the recorded US RF signals or images, for instance, by employing the cross-correlation algorithm.
(m) Similar to the previous algorithm, an elastic tumor is simulated by allocating the values of 1 Pa and 0.5 to the elastic modulus and Poisson's ratio of the tumor.
(n) The displacement values of the appointed points of tumor and elastic tumor at the selected instants are manipulated to extract the real elastic modulus of the tumor by the use of (23), as explicated in step (e).
(o) The sensitivity matrix is constructed in this step, as follows:
  (1) The set of estimated hyperelastic parameters,** C**, is assigned to the tumor. In the first iteration of the algorithm, the set of hyperelastic parameters computed for the tumor by the employment of the previously described method is taken into account. The displacement quantities of the selected points of tumor at every specified moment, **D**_*i*,**C**_ (in total,** D**), are regarded.
  (2) Based on the hyperelastic model considered for the tumor, the response of the tumor, after slightly altering each of the hyperelastic parameters, *C*_*j*_, for instance, about 0.1% of its value, is simulated; similarly, the displacement quantities of the same points of tumor at every determinate moment,** D**_i,Cj_, are extracted. The difference between the calculated displacement vectors at the corresponding step times,* ∂ ***D**_i_, is computed for all the specified moments.
  (3) The sensitivity matrix,** S**, [17, 18] is constructed as follows:
    (26) S=S11S12⋯S1qS21S22⋯S2q⋮⋮⋱⋮Sl1Sl2⋯Slq,Sij=∂Di∂Cj,∂Cj=μCj,
  where *q*, *μ*, and *l*, respectively, represent the number of hyperelastic parameters of the model, the amount of change in the hyperelastic parameters, and the number of consecutive step times when the responses of the tissue, i.e., the displacement quantities of some points of the tissue, have been computed.
(p) With regard to the Tikhonov regularization method, (27) [17, 18] is then used to compute the new hyperelastic parameters, **C**_est_, for the tumor,
  (27) $$\begin{array}{c} C_{est}={\left[\;S^{T}S+\alpha I\;\right]}^{-1}\left[\;S^{T}\left(\;Y_{real}-D\;\right)+S^{T}SC\;\right], \end{array}$$
where the parameter *α* represents the regularization parameter. On account of the achieved outcomes, there is no need to define the regularization parameter when the tissue displacement fields have been accurately measured from the recorded US RF signals and images.
(q) The estimated hyperelastic parameters, **C**_est_, are applied to calculate the tumor elastic parameter, *E*_sim_, and its error by comparing it with *E*_real_, as explained in step (h).
(r) As regards the estimated hyperelastic parameters, steps (o) to (q) are repeated until the error of the elastic parameter estimated for the tumor decreases below the defined tolerance value, as illustrated,
  (28) $$\begin{array}{c} \left\|\;E_{sim}^{k}-E_{real}\;\right\|\leq e, \end{array}$$
where *e* and *k*, respectively, represent the tolerance value and the number of iterations of the algorithm.

By reason of recording low-quality US RF signals or images, applying an imprecise motion tracking method, assigning inappropriate values to parameters of the motion tracking algorithm, for instance, the maximum lag in the cross-correlation algorithm, or other attributes, the displacements of selected points of tumor might not be measured correctly. It is required to determine an appropriate regularization parameter to converge to the tumor hyperelastic parameters while the iterative sensitivity-matrix based method is applied to the imprecise displacement measurements.

The errors of the elastic parameters estimated for the tumor could also be considered to allocate proper value to the regularization parameter. With reference to the attained outcomes, through assigning a very small value to the regularization parameter, the estimated hyperelastic parameters by manipulating the sensitivity-matrix based algorithm could converge to the real hyperelastic parameters of the tumor. Since it is assumed that no initial knowledge of the tumor and its mechanical features is available, similar to the two previously described algorithms, right value for the regularization parameter could also be determined regarding the errors of the elastic parameters estimated for the tumor.

Although obviously the meticulous simulation of the understudy tissue and its neighbors on the basis of the recorded predeformation US images, loading specifications, and boundary conditions by the use of the FEM software, Abaqus FEA, could significantly improve the results, the attained outcomes connote the stability of the estimates against the errors induced by the displacement measurement or other attributes. Furthermore, the simulation could be enhanced by the use of innovative strategies proposed to

- form three-dimensional (3D) volume data from two-dimensional (2D) US scans; hence Voxel-Based Methods (VBM), Pixel-Based Methods (PBM), and Function-Based Methods (FBM) could be applied [46, 47];
- compensate for the incomplete knowledge of the boundary conditions; notably, the problem of unknown conditions on part of the boundary has been solved by Hajhashemkhani et al. [6] using the Gauss-Newton method to minimize the cost function defined on the basis of the measured and calculated displacements.

## 3. Results and Discussion

### 3.1. Soft Tissue Simulation

**Figure 1:**
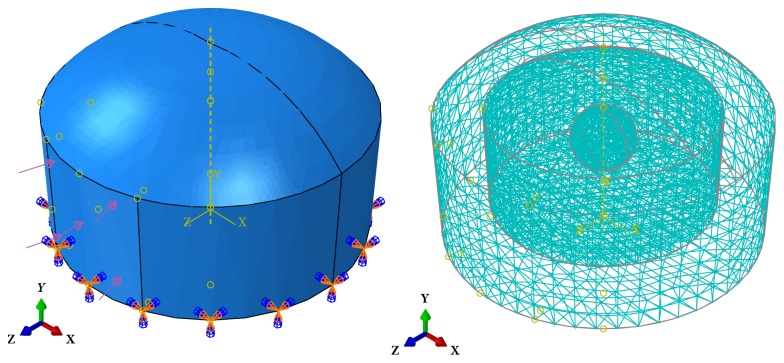
The load, boundary condition, and mesh applied to the simulated breast tissue which is composed of the normal fat and fibroglandular tissues and a spherical tumor.

With the aim of demonstrating the efficacy of the proposed method in estimating hyperelastic parameters of an unknown tissue, particularly an unidentified tumor in the breast, we have utilized the FEM software, Abaqus FEA, to simulate 3D breast tissue geometry. Three tissues, the fat, fibroglandular, and an interior spherical tumor, constitute the simulated breast tissue, which has been depicted in Figure 1. Figure 1 also represents the load applied to the medium and the defined boundary condition, i.e., Encastre. As previously explained, the iterative algorithms have been employed to estimate the parameters of three hyperelastic models, namely, the Mooney-Rivlin, Yeoh, and second-order polynomial models. The hyperelastic parameters allocated to the normal fat and fibroglandular breast tissues and benign and malignant breast tumors, i.e., fibroadenoma, invasive lobular carcinoma (ILC), and invasive mucous carcinoma (IMC), have been reported in Tables 1–4.

It is assumed that the mechanical parameters, i.e., the linear and nonlinear elastic parameters, are constant throughout each tissue type.  Table 1 demonstrates a set of elastic parameters and Mooney-Rivlin hyperelastic parameters of breast tissues which has been widely applied to simulate the breast [48–50]. The Yeoh and polynomial hyperelastic parameters, presented in Tables 2–4, have been reported by Samani and Plewes [44] (for the normal tissues) and O'Hagan and Samani [19] (for the benign and malignant tumors). An iterative method has been proposed by O'Hagan and Samani to estimate hyperelastic parameters of ex vivo breast tissue specimens from their responses to the low-frequency sinusoidal load. Their results confirm that the Yeoh and polynomial models are the hyperelastic models which conform more to the experimental data recorded from the breast tissues [19, 20].

In pursuance of the simulation of US RF signals and images by the use of the Field II US Simulation Program, as explained in Section 3.3, the positions of scatterers after applying the load to the simulated medium should be calculated. It is feasible to precisely compute their positions through augmenting the number of nodes in the medium. As depicted in Figure 1, 36303 4-node hybrid tetrahedron (C3D4H) elements with 7603 nodes have constituted the mesh of the simulated phantom. The accuracy of the simulation outcomes has been verified by the convergence analyses.

### 3.2. Estimation of Hyperelastic Parameters from Precise and Imprecise Displacement Measurements

The displacement distribution, specifically in the axial direction, in the in vivo tissue could be measured from the data recorded by the use of the standard medical imaging systems such as the US or magnetic resonance imaging (MRI) system in a noninvasive way; therefore, in view of estimating hyperelastic parameters of the tumor, we have employed the axial displacement values of some points of tissue while the tissue is responding to the ramp stimulation. With the purpose of ignoring the inertia effect, the rate of increase in the applied load is considered low.

With regard to the displacement values of several points of tumor at some step times, the estimates of parameters of the Mooney-Rivlin, Yeoh, and second-order polynomial models, achieved by the use of the iterative methods explicated in Section 2.2, have briefly been represented in Tables 5–10. The results have been reached by applying the displacements of maximum fifteen points of tumor in maximum twelve consecutive instants (with the difference of 0.25 s between the step times). Higher values have been considered for the second-order polynomial model with five indeterminate parameters, particularly while the displacement measurements were inexact.

The results associated with estimating the hypothetical and real elastic parameters and hyperelastic parameters of the Mooney-Rivlin model for the normal and abnormal breast tissues by the use of the proposed iterative methods have been represented in Tables 5 and 6. The outcomes of the estimation of hypothetical and real hyperelastic parameters of the Yeoh model for the benign and malignant breast tumors, i.e., the fibroadenoma and ILC, by applying the suggested algorithms have been summarized in Tables 7, 8, and 9.  Table 10 demonstrates the capability of the recommended techniques in properly computing the hypothetical and real hyperelastic parameters of the second-order polynomial model based on the response of the malignant IMC tumor to the ramp excitation.

The calculation of displacement distributions in the stimulated breast tissue from the pre- and postdeformation RF signals simulated by the use of the Field II US Simulation Program [51] (A MATLAB® toolbox for US field simulation) has implied the necessity to consider the errors of displacement measurements.  Table 11 indicates the competence of two propounded methods, i.e., the iterative stress-strain relation-based and sensitivity-matrix based algorithms, in properly computing the hypothetical and real hyperelastic parameters from the inexact estimates of tumor displacement fields.

### 3.3. Simulation of US RF Signals and Images

The Field II US Simulation Program [51] has been applied to simulate the pre- and postdeformation RF signals (on the basis of the response of the breast tissue to the applied load) to appraise the errors of displacement measurements. While the RF signals of breast tumors surrounded by the normal fat and fibroglandular tissues were being simulated, zero acoustic impedance was allocated to the abnormal breast tissues. In the Field II US Simulation Program, the properties selected to model the probe array and simulate the US RF signals have been considered as follows:

- Linear array,
- An array with 64 active elements,
- Transducer center frequency: 5 MHz,
- Sampling frequency: 60 MHz,
- Transmit focus (in depth): 50 mm,
- Pitch of probe array (i.e., element's width): 0.44 mm, that is equal to the wavelength,
- Element's height: 5 mm,
- Element's kerf: 0.03 mm,
- Number of scan lines in the image: 256 lines (i.e., lateral spatial spacing of 0.16 mm).

**Figure 2:**
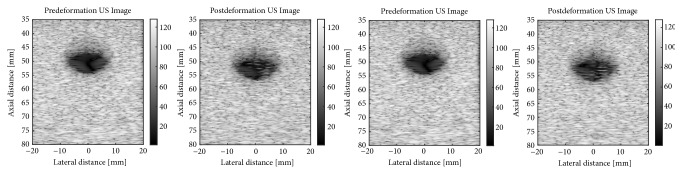
The B-mode images of the breast tissue constructed from the RF signals simulated on the basis of the tissue states at t=0.00 s and t=7.50 s (left) and t=0.00 s and t=9.00 s (right), while responding to the ramp excitation.

In addition, the positions of scatterers in each deformation state have been calculated by linearly interpolating the displacements of the adjacent nodes computed through the FEM software, Abaqus FEA. To illustrate, two sets of B-mode images of the breast tissue, i.e., the pre- and postdeformation B-mode images, constructed from the RF signals associated with the times of 7.50 s and 9.00 s after starting to apply the ramp excitation, have been represented in Figure 2.

The gold standard of displacement estimation from the US RF signals, i.e., the cross-correlation algorithm, has been applied to evaluate the errors of displacement measurements. The displacement distributions in the tumor at the selected step times have been calculated from the pre- and postdeformation RF signals. In comparison with the displacement measurement from the US images, more precise estimate of tissue displacement field with higher spatial resolution is obtained by utilizing the RF signals.

In view of declining the computation time, which significantly raises by the use of the RF signals due to their higher sampling rate, two tactics have been employed:

1. Through defining maximum lag in regard to the estimated displacements of adjacent regions, i.e., preceding samples and lines, each search area (the number of lags considered in specifying the maximum of the cross-correlation function for two corresponding windows of the RF signals) was reduced considerably [52].
2. The computational cost of the cross-correlation algorithm was appreciably declined by eliminating repeated calculations with the help of precomputed sum tables [53].

## 4. Conclusions

The correct identification of benign and malignant tumors in the breast through their nonlinear elastic parameters by the use of a noninvasive and nondestructive method has been intended in this paper. With respect to the principles of elastography technique, two successive iterative algorithms, founded on

1. the relation between stress, strain, and the parameters of a hyperelastic model,
2. the sensitivity matrix, which correlates the changes in the displacement field in the tissue to the variations of hyperelastic parameters,
3. the estimation of displacement and strain fields in the tissue from the recorded US RF signals and images using the motion tracking techniques,

(as explained in the Materials and Methods section of the paper), have been utilized to precisely estimate the parameters of three hyperelastic models,

- the Mooney-Rivlin model, i.e., the parameters *C*_10_ and *C*_01_ of (10),
- the Yeoh model, i.e., the parameters *C*_10_, *C*_20_, and *C*_30_ of (11),
- the second-order polynomial model, i.e., the parameters *C*_10_, *C*_20_, *C*_11_, *C*_02_, and *C*_01_ of (12).

The exact/inexact displacement values of limited points (at restricted instants) of the tumor excited by the ramp stimulus have been applied to calculate the parameters.

The dependency of the proposed method to the displacement and strain quantities warrants its competence as a noninvasive stratagem. The displacement and strain fields in the in vivo tissue could be computed from the recorded US RF signals or images by the employment of motion tracking approaches, which have been typically classified into three categories [53],

- Phase-domain methods,
- Time-domain (1D) or Space-domain (2D) methods,
- Spline-based methods.

The reliance of a method to precise values of deformation variables except or further than the displacement or strain, as the ones proposed by Omidi et al. [54], Roy and Desai [55], Liu et al. [56], Boonvisut and Çavuşoğlu [57], and Wang et al. [58], to mention a few, prevents the technique from being considered an elasticity imaging approach with the capability to noninvasively depict the nonlinear elastic features of in vivo tissues.

The inadequacy of the displacement-based techniques, for instance, two methods proposed by Mehrabian and Samani [59–61] and Hajhashemkhani and Hematiyan [17, 18], necessitates the improvement of the tactics or the introduction of novel strategies for the hyperelastic elastography. The main defects of the aforementioned methods (as some of them have been assessed in [62]) are as follows:

1. the dependency of the defined coefficient matrix in the former method to the precise displacement measurements of a great number of adjacent points inside the medium and the reliance of the latter method to the displacement values of some boundary points of the tumor, which might not be accurately extracted from the recorded data, e.g., the US RF signals or images,
2. the necessity to have initial knowledge of the tumor in order to (a) consider proper initial guesses for the hyperelastic parameters to initiate the algorithms and (b) be assured of converging to the main hyperelastic parameters, specifically on account of the defined criteria to stop the algorithms,
3. the requisite to employ appropriate regularization methods and parameters, for example, as indicated by Mehrabian and Samani, the truncated singular value decomposition (SVD), Tikhonov regularization, and Wiener filtering techniques [59–61],

which have been rectified in the proposed method. The higher error of displacement estimates in the boundary region has been discussed in [63–65].

It has been assumed that no primary knowledge of the tumor is accessible except the exact or even approximate measurements of displacement and strain fields in the tumor. With consideration of the estimated hyperelastic parameters, the minimum error of the elastic parameters calculated for the tumor, while they are compared with the main elastic parameter computed for the tumor from the estimated displacement fields in the tumor, is the principal criterion for the choice of the best estimates of hypothetical and real hyperelastic parameters.

Thanks to the defined criterion, the repeated manipulation of the stress-strain relation of the hyperelastic model results in

- converging to the proper initial estimates of the parameters of the hyperelastic model; therefore, the possibility of inaccurately estimating the hyperelastic parameters of the understudy tissue declines to zero;
- significantly reducing the number of iterations of the iterative sensitivity-matrix based method, i.e., the computational cost of the real hyperelastic parameter estimation algorithm.

In pursuance of fulfilling the breast hyperelastic elastography, the propounded technique will be applied to estimate the nonlinear elastic constants of in vivo breast abnormalities from the recorded US RF signals and images in the future research.

## Figures and Tables

**Table 1:** The linear and nonlinear (Mooney-Rivlin) elastic parameters of normal and abnormal breast tissues [48].

Hyperelastic & Elastic Parameters			
Breast Tissues	*C*_10_ (Pa)	*C*_01_ (Pa)	*E* (kPa)

Fat	2000	1333	20
Fibroglandular	3500	2333.3	35
Tumor	10000	6667	100

**Table 2:** The 2^nd^-order polynomial hyperelastic parameters of normal breast tissues, namely, the fat and fibroglandular tissues [44].

Hyperelastic Parameters					
Normal Breast Tissues	*C*_10_ (Pa)	*C*_01_ (Pa)	*C*_20_ (Pa)	*C*_11_ (Pa)	*C*_02_ (Pa)

Fat	310	300	3800	2250	4720
Fibroglandular	330	280	7720	4490	9450

**Table 3:** The Yeoh hyperelastic parameters of abnormal breast tissues, namely, the fibroadenoma and invasive lobular carcinoma (ILC) [19].

Hyperelastic Parameters			
Benign & Malignant Breast Tumors	*C*_10_ (Pa)	*C*_20_ (Pa)	*C*_30_ (Pa)

Fibroadenoma	1190	20400	0
ILC	2066	2045	998

**Table 4:** The 2^nd^-order polynomial hyperelastic parameters of abnormal breast tissue, namely, the invasive mucous carcinoma (IMC) [19].

Hyperelastic Parameters					
Malignant Breast Tumor	*C*_10_ (Pa)	*C*_01_ (Pa)	*C*_20_ (Pa)	*C*_11_ (Pa)	*C*_02_ (Pa)

IMC	1340	1340	9830	9833	6090

**Table 5:** The hypothetical and real hyperelastic parameters of the breast tumor estimated for the Mooney-Rivlin model by the use of the proposed iterative stress-strain relation-based and sensitivity-matrix based algorithms.

Hypothetical Hyperelastic Parameters			Real Hyperelastic Parameters		
Hypothetical Hyperelastic & Elastic Parameters	First Estimates	Second Estimates	Real Hyperelastic Parameters	Second Iteration^*∗*^	Fifth Iteration

*E*_real_ (kPa)	9.89728773e+04		Hypothetical *C*_10_ (Pa)	1.396611e+04	
Error of *E*_real_ (%)	1.0271		Hypothetical *C*_01_ (Pa)	2.72700e+03	
*C*_10_ (Pa)	1.40034876e+04	1.39661106e+04	*C*_10_ (Pa)	1.00062196e+04	0.99996187e+04
*C*_01_ (Pa)	2.6837125e+03	2.72700048e+03	*C*_01_ (Pa)	6.6604884e+03	6.6673814e+03
Error of *C*_10_ (%)	40.0349	39.6611	Error of *C*_10_ (%)	0.0622	0.0038
Error of *C*_01_ (%)	59.7463	59.0970	Error of *C*_01_ (%)	0.0977	0.0057
*E*_sim_ (kPa)	9.89728999e+04	9.89736850e+04	*E*_sim_ (kPa)	9.89728358e+04	9.89728776e+04
Error of *E*_sim_ (%)	2.2868e-05	8.1606e-04	Error of *E*_sim_ (%)	4.1895e-05	2.8726e-07

^*∗*^Precise estimates have been achieved in the primary iterations of the iterative sensitivity-matrix based algorithm by the use of the computed hypothetical hyperelastic parameters.

**Table 6:** The hypothetical and real hyperelastic parameters of the normal breast tissue, namely, the fibroglandular tissue, estimated for the Mooney-Rivlin model using the proposed iterative stress-strain relation-based and sensitivity-matrix based algorithms.

Hypothetical Hyperelastic Parameters			Real Hyperelastic Parameters (In regard to first estimates of hypothetical hyperelastic parameters)		
Hypothetical Hyperelastic Parameters	First Estimates	Second Estimates	Real Hyperelastic Parameters	Third Iteration^*∗*^	Fifth Iteration

*C*_10_ (Pa)	1.5851138e+03	1.0814951e+03	*C*_10_ (Pa)	3.5088899e+03	3.4997360e+03
*C*_01_ (Pa)	26.5302	7.746264e+02	*C*_01_ (Pa)	2.3235739e+03	2.3335629e+03
Error of *C*_10_ (%)	54.7110	69.1001	Error of *C*_10_ (%)	0.2540	0.0075
Error of *C*_01_ (%)	98.8630	66.8013	Error of *C*_01_ (%)	0.0042	0.0001
Error of *E*_sim_ (%)	2.0536	1.9088	Error of *E*_sim_ (%)	3.1787e-04	1.6123e-07

^*∗*^Precise estimates have been obtained in the primary iterations of the iterative sensitivity-matrix based algorithm by making use of the calculated hypothetical hyperelastic parameters.

**Table 7:** The hypothetical hyperelastic parameters of the malignant tumor, namely, the ILC, in the breast estimated for the Yeoh model using the suggested iterative stress-strain relation-based algorithm.

Hypothetical Hyperelastic Parameters^*∗*^		Estimated Hyperelastic Parameters	Estimates of Previous Iteration^*∗*^	Estimates of Next Iteration^*∗*^
*C*_10_ (Pa)	0.18034160e+04	*C*_10_ (Pa)	1.7951433e+03	1.7934292e+03
*C*_20_ (Pa)	0.15985308e+04	*C*_20_ (Pa)	3.0443848e+03	3.1841804e+03
*C*_30_ (Pa)	6.63359617e+04	*C*_30_ (Pa)	0.0002843e+03	0.0002742e+03
Error of *C*_10_ (%)	0.0127098e+03	Error of *C*_10_ (%)	13.1102	13.1932
Error of *C*_20_ (%)	0.0218322e+03	Error of *C*_20_ (%)	48.8697	55.7056
Error of *C*_30_ (%)	6.5468899e+03	Error of *C*_30_ (%)	99.9715	99.9725
Error of *E*_sim_ (%)	0.2512	Error of *E*_sim_ (%)	0.2593	0.2611

^*∗*^In consideration of the minimum error of the estimates of tumor elastic modulus, the hypothetical hyperelastic parameters have been chosen. The parameters achieved in the previous and next iterations do not straightly converge to the real hyperelastic parameters of the tumor by the use of the iterative sensitivity-matrix based algorithm.

**Table 8:** The real hyperelastic parameters of the malignant tumor, namely, the ILC, in the breast estimated for the Yeoh model using the iterative sensitivity-matrix based algorithm.

Real Hyperelastic Parameters		Third Iteration^*∗*^		Seventh Iteration	
*C*_10_ (Pa)	Error of *C*_10_ (%)	2.0660258e+03	0.0012	2.0660161e+03	0.0008
*C*_20_ (Pa)	Error of *C*_20_ (%)	2.0398643e+03	0.2511	2.0433674e+03	0.0798
*C*_30_ (Pa)	Error of *C*_30_ (%)	1.1566597e+03	15.8978	1.0457290e+03	4.7825

^*∗*^Precise estimates (esp. for *C*_10_ and *C*_20_) have been achieved in the primary iterations of the iterative sensitivity-matrix based algorithm by using the calculated hypothetical hyperelastic parameters (reported in Table 7).

**Table 9:** The hypothetical and real hyperelastic parameters of the benign tumor, namely, the fibroadenoma, in the breast estimated for the Yeoh model using the proposed iterative stress-strain relation-based and sensitivity-matrix based algorithms.

Hypothetical Hyperelastic Parameters		Real Hyperelastic Parameters		
Hypothetical Hyperelastic Parameters	Estimates	Real Hyperelastic Parameters	First Iteration^*∗*^	Seventh Iteration

*C*_10_ (Pa)	1.6801636e+03	*C*_10_ (Pa)	1.1862355e+03	1.1900172e+03
*C*_20_ (Pa)	2.8495611e+03	*C*_20_ (Pa)	1.99515281e+04	2.03980890e+04
*C*_30_ (Pa)	0.0002326e+03	*C*_30_ (Pa)	45.4462	60.9599
Error of *C*_10_ (%)	41.1902	Error of *C*_10_ (%)	0.3163	0.0014
Error of *C*_20_ (%)	86.0316	Error of *C*_20_ (%)	2.1984	0.0094
Error of *E*_sim_ (%)	0.6687	Error of *E*_sim_ (%)	6.4873e-03	2.7053e-05

^*∗*^Precise estimates have been obtained in the primary iterations of the iterative sensitivity-matrix based algorithm by the use of the computed hypothetical hyperelastic parameters.

**Table 10:** The hypothetical and real hyperelastic parameters of the malignant tumor, namely, the IMC, in the breast estimated for the 2^nd^-order polynomial model using the proposed iterative stress-strain relation-based and sensitivity-matrix based algorithms.

Hypothetical Hyperelastic Parameters			Real Hyperelastic Parameters (In regard to the first set of estimated hypothetical hyperelastic parameters)		
Hypothetical Hyperelastic Parameters	First Estimates	Second Estimates	Real Hyperelastic Parameters	First Iteration^*∗*^	Seventh Iteration

*C*_10_ (Pa)	1.3708224e+03		*C*_10_ (Pa)	1.3339340e+03	1.3400020e+03
*C*_01_ (Pa)	1.3098539e+03		*C*_01_ (Pa)	1.3460369e+03	1.3399977e+03
*C*_20_ (Pa)	1.14572903e+04	1.23904893e+04	*C*_20_ (Pa)	1.01126656e+04	0.99257766e+04
*C*_11_ (Pa)	1.12487168e+04	1.22630919e+04	*C*_11_ (Pa)	9.7807305e+03	9.6482256e+03
*C*_02_ (Pa)	0.56449179e+04	0.61441807e+04	*C*_02_ (Pa)	5.8167728e+03	6.1791597e+03
Error of *C*_10_ (%)	2.3002		Error of *C*_10_ (%)	0.4527	0.0001
Error of *C*_01_ (%)	2.2497		Error of *C*_01_ (%)	0.4505	0.0002
Error of *C*_20_ (%)	16.5543	26.0477	Error of *C*_20_ (%)	2.8755	0.9743
Error of *C*_11_ (%)	14.3976	24.7136	Error of *C*_11_ (%)	0.5316	1.8791
Error of *C*_02_ (%)	7.3084	0.8897	Error of *C*_02_ (%)	4.4865	1.4640
Error of *E*_sim_ (%)	5.7883e-04	7.7111e-04	Error of *E*_sim_ (%)	9.7628e-06	2.1205e-07

^*∗*^Precise estimates have been attained in the primary iterations of the iterative sensitivity-matrix based algorithm by making use of the computed (first set of) hypothetical hyperelastic parameters.

**Table 11:** The hypothetical and real hyperelastic parameters of the breast tumor estimated for the Mooney-Rivlin model from the imprecise displacement measurements, with 2%, 5%, and 10% errors, by the use of proposed tactics.

Hypothetical Hyperelastic Parameters estimated by the use of the iterative stress-strain relation-based algorithm					
	Hypothetical Hyperelastic Parameters		Hypothetical Hyperelastic Parameters	Hypothetical Hyperelastic Parameters	
	Displacement Error of 2%		Displacement Error of 5%	Displacement Error of 10%	

*E*_real_ (kPa)	9.90977910e+04		9.92857540e+04	9.96006152e+04	
Error of *E*_real_ (%)	0.9022		0.7142	0.3994	
*C*_10_ (Pa)	1.05936892e+04		7.9382924e+03	1.04180049e+04	4.9146920e+03
*C*_01_ (Pa)	7.0947542e+03		1.13425870e+04	7.4619495e+03	1.76362097e+04
Error of *C*_10_ (%)	5.9369		20.6171^*∗*^	4.1800	50.8531^*∗*^
Error of *C*_01_ (%)	6.4160		70.1303^*∗*^	11.9236	1.645299e+02^*∗*^
*E*_sim_ (kPa)	9.90992665e+04		9.92811200e+04	9.91221342e+04	9.95989786e+04
Error of *E*_sim_ (%)	0.0015		0.0047	0.4804	0.0016

Real Hyperelastic Parameters estimated by the use of the iterative sensitivity-matrix based algorithm					

	Real Hyperelastic Parameters		Real Hyperelastic Parameters	Real Hyperelastic Parameters	
	Displacement Error of 2%		Displacement Error of 5%	Displacement Error of 10%	

Iteration	First Iteration^*∗*^	Second Iteration^*∗*^	Second Iteration^*∗*^	First Iteration^*∗*^	Forth Iteration

*α*	3.0000e-19	5.0000e-19	10.0000e-19	2.0000e-18	2.0000e-18
*C*_10_ (Pa)	1.00470535e+04	9.5997294e+03	9.9363293e+03	9.4346723e+03	0.98151514e+04
*C*_01_ (Pa)	6.4888826e+03	7.0114736e+03	6.6044865e+03	6.8200699e+03	6.5820232e+03
Error of *C*_10_ (%)	0.4705	4.0027	0.6367	5.6533	1.8485
Error of *C*_01_ (%)	2.6716	5.1668	0.9377	2.2959	1.2746

^*∗*^Precise estimates of real hyperelastic parameters have been achieved in the primary iterations by using the iterative sensitivity-matrix based algorithm as the result of determining appropriate regularization parameter even in the conditions where the errors of the estimates of hypothetical hyperelastic parameters were high.

## Data Availability

The data used to support the findings of this study are available from the corresponding author upon request.
